# Association of polymorphisms in *Pit-1* gene with growth and feed efficiency in meat-type chickens

**DOI:** 10.5713/ajas.18.0173

**Published:** 2018-07-26

**Authors:** Sihua Jin, Tingting He, Lei Yang, Yucui Tong, Xingyong Chen, Zhaoyu Geng

**Affiliations:** 1 College of Animal Science and Technology, Anhui Agricultural University, Hefei 230036, China

**Keywords:** Chicken, *Pit-1*, Single Nucleotide Polymorphism, Feed Conversion Ratio, Association

## Abstract

**Objective:**

The pituitary specific transcription factor-1 (*Pit-1*) gene is responsible for pituitary development and growth hormone expression and is regarded as a pivotal candidate gene for growth and production in chickens. Therefore, the aim of this study was to investigate the association of *Pit-1* polymorphisms with growth and feed efficiency traits in yellow meat-type chickens.

**Methods:**

In the present study, five single nucleotide polymorphisms (SNPs) of *Pit-1* were selected and genotyped by high-throughput matrix-assisted laser desorption-ionization time-of-flight mass spectrometry in 724 meat-type chickens.

**Results:**

Association analysis showed that rs13687126 of *Pit-1* was strongly associated with body weight gain (BWG) and feed intake (FI) (p<0.05), and that rs13687128 was significantly correlated with body weight at 70 days of age (BW70), BWG and feed conversion ratio (FCR) (p<0.05). SNP rs13905622 was strongly related to BW70 and FCR (p<0.05). Furthermore, birds with the GG genotype of rs13687126 had larger BWG and FI than those with the AG genotype (p<0.05). Individuals with the TT genotype of rs13687128 were significantly higher BW70 and BWG than those of the CT and CC genotype, while FCR was just the opposite (p<0.05). For rs13905622, the AA chickens showed strongly larger BW70 and lower FCR compared with the AT and TT chickens (p<0.05). Additionally, an ACA haplotype based on rs13687126, rs13687128, and rs13905622 had significant effects on BW70 and FCR (p<0.05).

**Conclusion:**

Our studies thus provide crucial evidence for the relationship between polymorphisms of *Pit-1* and growth and feed efficiency traits which may be useful for meat-type chicken breeding programs.

## INTRODUCTION

In the past decades, attention has paid to growth and feed efficiency traits for farm animals because feed cost is an important factor of profitability in the animal husbandry. In modern poultry industry, feed efficiency exhibits a competitive position against other animal protein sources, and superior efficiency indicates less requirement on global feed resources [1]. Meanwhile, low efficiency birds produce excess excreta resulting in a negative impact on environment [2]. In recent years the progress on optimizing diet compositions had positive effects on the feed efficiency in meat-type chickens. Nonetheless, further improvement can be gained in feed efficiency through genetics and genomic selection. Therefore, it is important for producers and breeders to select more efficient birds in broiler breeding programs, and thus improve profitability.

The pituitary specific transcription factor-1 (*Pit-1*, or POU class 1 homeobox 1) gene belongs to a family of transcriptional regulators and is involved in differentiation and proliferation of cells by mediating the transcription of growth hormone, prolactin (*PRL*), thyroid stimulation hormone-β subunit and *Pit-1* itself in animals [3]. The *Pit-1* gene is also responsible for the development of the anterior pituitary [4], inducing the differentiation of hepatic progenitor cells into PRL-producing ones [5] and delaying adrenarche in humans [6]. Thus, it plays a crucial role in regulating the growth and development of animals. Mutations in the *Pit-1* gene are significantly associated with human disorders [7]. Previous studies showed that *Hinf*I polymorphism in exon 6 of the *Pit-1* gene was strongly correlated with protein percentage, and the AB genotype had higher milk protein percentage than those of the AA and BB genotype in Holstein cattle [8]. It was reported that individuals with the AA genotype of rs80904061 in intron 4 of the *Pit-1* gene had significantly lower daily feed intake (FI), feed to gain ratio and number of days to finishing than those with the BB genotype in pigs raised in Poland [9]. Tang et al [10] also demonstrated that polymorphisms in *Pit-1* intron 5 were significantly related to body weight (BW), average daily gain and chest girth at 6, 12, 18, and 24 months of age, and allele A may be an advantageous one for growth traits in Chinese cattle. Currently, in accordance with chicken genome assembly (*Gallus_gallus* 5.0), the *Pit-1* gene is genetically mapped on chromosome 1 and includes 7 exons and 6 introns spanning 14.0 kb in the proximity of quantitative trait loci (QTL) associated with growth and development (https://www.animalgenome.org/cgi-bin/QTLdb/) [11]. Another study found that the single nucleotide polymorphism (SNP) of MR5 in the *Pit-1* gene was significantly related to BW at 21 and 35 days of age, and it had significant effects on average daily gain at 0 to 4 weeks, thus C allele was dominant for chicken growth [12]. Xu et al [13] found that g.96217999 T> C genotype was strongly correlated with BW in SD03 strain of Chinese native chickens.

To date, few studies on the relationship between polymorphisms in the *Pit-1* gene and growth and feed efficiency traits had been reported in yellow meat-type chickens. In China, meat-type broilers are mainly classified into two categories, the fast growing white feathered broilers and various yellow feathered indigenous chickens. Therefore, the objective of this study was to examine the association of *Pit-1* polymorphisms with BW, body weight gain (BWG), FI, and feed conversion ratio (FCR) in yellow meat-type chicken populations.

## MATERIALS AND METHODS

### Ethics statement

All animal experiments were conducted according to the Regulations and Guidelines for Experimental Animals established by the Ministry of Science and Technology (Beijing, China, revised in 2004) and approved by the Institutional Animal Care and Use Committee of Anhui Agricultural University (approval ID: IACUC-20101020). All experiment procedures were strictly performed in accordance with the regulations and recommendations of this committee, and all efforts were to relieve suffering of the chickens.

### Chicken populations and phenotype measurements

In the present study, the same chicken strains as reported by Jin et al [14] were chosen as the experimental populations. Briefly, the population was composed of 796 pedigreed males from the 22nd generation of two yellow meat-type chicken lines N301 and N202 with complete pedigree data (G_20_ to G_22_), comprising 461 birds from the N301 and 335 chicks from the N202 strain. The two strains were chosen as closed populations in accordance with appearance, growth and carcass traits in every generation and selected as important genetic sources for future indigenous chicken breeding schemes. All birds were obtained from a single hatch, wing-banded at hatch, raised indoors and handed in a routine way.

All experimental diets were formulated based on the nutrient requirements and levels of meat-type chickens of China (criterion number: NY/T 33-2004) and the ingredient composition and analyzed nutrient levels are listed in Table 1. The same feeding regime was offered by Wens Nanfang Poultry Breeding Co. Ltd., Guangdong, China. Feed and water were provided *ad libitum* during the entire experimental period. A proper schedule for vaccination was followed for all birds, such as Marek’s disease vaccine on day 1, New Castle disease vaccine on day 7, infectious bursal disease vaccine on day 14 and day 24, and New Castle disease vaccine on day 28. Cooling facilities during the summer and proper lighting were carried out to provide the a comfortable environment for the chickens to achieve their optimum potential.

At 42 days of age, all birds were randomly transferred into individual cages with the size of 25×40×45 cm and particular troughs adopted for measuring respective FI. Body weight at 49 (BW49) and 70 days of age (BW70), and FI in the interval were recorded individually. The BWG and FCR were calculated based on BW and FI from 49 to 70 days of age. However, 72 chickens were removed because of missing phenotypic or genotypic information. Finally, a total of 724 chickens, including 439 chickens from the N301 stain and 285 birds from the N202 line, were used for further association analysis of *Pit-1* polymorphisms with growth and feed efficiency traits in meat-type chickens.

### SNPs selection

The SNPs in the *Pit-1* gene were obtained from Ensembl Data mining tool BioMart (http://www.ensembl.org/), NCBI SNP database (http://www.ncbi.nlm.nih.gov/) and UCSC Genome Browser database (http://genome.ucsc.edu/cgi-bin/hgGateway). The DNAMAN 7.0 (Lynnon Biosoft, Vaudreuil, QC, Canada) and BLAST program at the NCBI database were used to ensure all selected SNP sequences were non-homologous compared with other genome sequences. All SNPs harbored an rs# number which enabled information to be accessed on dbSNP. Finally, 5 SNPs in the *Pit-1* gene were selected for further association analysis.

### Genotyping and quality control

Blood samples were collected from brachial veins of chickens by standard venipuncture and transferred into blood collection tubes containing acid citrate dextrose anticoagulant at −20°C prior to DNA isolation. Genomic DNA was isolated from whole blood samples using a TIANamp Blood DNA Kit (Tiangen Biotech Co. Ltd., Beijing, China) according to the manufacturer’s instructions. The relative integrity of genomic DNA was detected by 1.5% agarose gel electrophoresis. DNA concentration was quantified by a NanoDrop2000 spectrophotometer (ThermoFisher Scientific, Waltham, MA, USA) and the final DNA concentrations were 2 to 10 ng/μL.

The SNP genotyping of 724 samples was performed using matrix-assisted laser desorption-ionization time-of-flight mass spectrometry (MALDI-TOF MS) based on the Mass ARRAY iPLEX Platform (Sequenom, San Diego, CA, USA). In these chip analyses, we randomly designed 5 repeats to guarantee the reliability of these genotyping methods. Two polymerase chain reaction primers and one extension primer were designed by Assay Design 3.1 software (Sequenom, USA) for each SNP, as shown in Table 2. The SNPs with a minor allele frequency <1% and genotype call rate <90% through all individuals were removed for further analysis.

### Linkage disequilibrium analysis and haplotype reconstruction

The Haploview software was used to determine the linkage disequilibrium (LD) between several SNPs in the *Pit-1* gene [15]. Lewontin D′ statistic >0.8 and squared correlation parameter r2>1/3 revealed adequately strong LD [16]. The haplotypes for SNPs in strong LD were analyzed by Phase software (v2.1.1, http://stephenslab.uchicago.edu/software.html).

### Statistical analysis

The genotypes and allele frequencies of SNPs in the *Pit-1* gene and Hardy-Weinberg equilibrium test were performed by FREQ procedure and chi-square fitness test of SAS version 9.4 (SAS Institute Inc., Cary, NC, USA). The SNPs that were inconsistent with Hardy-Weinberg equilibrium were discarded before further analysis. The association of remaining SNPs or haplotypes with growth and feed efficiency traits was performed using the generalized linear mixed model procedure of SAS 9.4 according to the following model:

$${\text{Y}}_{\text{ijk}}=\mu +{\text{S}}_{\text{i}}+{\text{G}}_{\text{j}}+{\text{F}}_{\text{k}}+{\text{e}}_{\text{ijk}}$$

Where Y_ijk_ represents the observed values of the traits, μ is the population mean, S_i_ is the fixed effect of strain, G_j_ is the fixed effect of SNP genotype or haplotype, F_k_ is the random effect of family, and e_ijk_ are the residuals. A probability level of p value ≤0.05 was considered to be significant.

## RESULTS AND DISCUSSION

The *Pit-1* gene is a member of the POU domain gene family and serves as an important tissue specific transcription factor mainly expressed in the anterior pituitary [3]. It is auto-regulated during expression and mediates gene expression especially in somatotrophs, lactotrophs, and thyrotrophs [17], which play pivotal roles in the growth of the pituitary and hormone secretion in animals [18]. In addition, the *Pit-1* gene was considered as an important candidate gene since it was genetically located on GGA1 which was near the QTL region controlling growth and development in chickens [19]. Accordingly, the association of the *Pit-1* gene polymorphisms with growth and feed efficiency traits were investigated to determine which could contribute to breeding high growth rate and superior efficiency birds. The basic statistics of growth and feed efficiency traits in two chicken strains were further analyzed and shown in the Supplementary Table 1. In this study, 5 SNPs in the *Pit-1* gene in the proximity of QTL area influencing BW and FCR were chosen and genotyped by MALDI-TOF MS in yellow meat-type chicken lines. All SNPs conformed to Hardy-Weinberg equilibrium and were further observed as polymorphic with a genotype call rate >90% and minor allele frequency >1%, as listed in Table 3.

Although many studies had investigated the molecular structure and the relationship between polymorphisms of *Pit-1* and growth and production traits in animals [8,9], few studies on associations of *Pit-1* polymorphisms with growth and feed efficiency traits had been reported in yellow meat-type chickens. In the present study, association analysis showed that three SNPs in the *Pit-1* gene were significantly related to some growth and feed efficiency traits in chickens (Table 4). Rs13687126 of the *Pit-1* gene was strongly associated with BWG and FI (p<0.05), and that rs13687128 was significantly correlated with body weight at 70 d of age (BW70), BWG and FCR (p<0.05). SNP rs13905622 was strongly related to BW70 and FCR (p<0.05). Recent study has demonstrated that g.9432T>C polymorphism in intron 5 of the *Pit-1* gene was strongly related to BW at 2 weeks, weight gain from hatch to 2 weeks, and weight gain from 16 to 18 weeks in Korean native chicken [20]. Selvaggi et al [21] reported that polymorphism of Pit-1/*Hinf*I was strongly related to growth traits and Pit-1/*Taq*I was significantly associated with early development from birth to weaning in Podolica young bulls.

In addition, the LD map of the haplotypes identified in the current study revealed a high linkage block among rs13687126, rs13687128, and rs13905622 of the *Pit-1* gene (Supplementary Figure S1). In addition, 7 haplotypes with a frequency >1.0% in the present population contributed to 98.68% of total haplotypes scored. They were H1 (ACA: 48.35%), H2 (ACT: 8.08%), H3 (ATA: 6.01%), H4 (ATT: 9.98%), H5 (GCA: 3.01%), H6 (GCT: 4.41%), and H7 (GTA: 19.02%), respectively (Table 5). Further association analysis showed that the ACA haplotype was significantly associated with BW70 and FCR (p<0.05, Table 4). It was demonstrated that the haplotypes of *Pit-1* gene had a significant effect on BW at 7 weeks of age in PB-1 chickens and were strongly associated with growth rate from 0 to 2 weeks of age in broiler strain and between 0 to 2 and 6 to 7 weeks of age in IWI layer strains [22]. Previous research also revealed that haplotypes in the *Pit-1* gene were strongly correlated with BW at 28 days of age, hatch weight, and shank diameters in an F2 chicken resource population of chickens [12].

Currently, it is well documented that polymorphisms of the *Pit-1* gene were significantly associated with dwarfism in humans [7], BW in pigs [23], and growth traits in chickens [22]. In the current study, birds with the GG genotype of rs13687126 had larger BWG and FI than those with the AG genotype (p< 0.05, Table 6). Birds with the TT genotype of rs13687128 had significantly higher BW70 and BWG than those of the CT and CC genotype, while FCR was just the opposite (p<0.05, Table 6). The genotype TT therefore might be a dominant one and allele T might be a dominant allele for growth and feed efficiency in chickens. For rs13905622, individuals with the AA genotype showed strongly larger BW70 and lower FCR compared with the AT and TT chickens (p<0.05, Table 6). Thus, A allele of rs13905622 might be advantageous for chicken growth and feed efficiency in yellow meat-type chickens. It was reported that individuals with the AA genotype in exon 3 of the *Pit-1* gene showed significantly higher weaning BW than those of the GG genotype in two Iranian sheep breeds [24]. Another study showed that the TT birds of g. 96219442 C>T of the *Pit-1* gene had significantly lower BW in the SD03 chicken strain compared with those of the CC birds [13]. However, there is little published literature about the relationship between *Pit-1* polymorphisms and FCR and FI in yellow meat-type chickens. Our results also showed that the TT birds of rs13687128 had lower FCR than those of the CC and CT chickens, and that individuals with the AA genotype of rs13905622 also have lower FCR compared with those of the AT and TT genotypes in our chicken populations (p<0.05). Therefore, our findings will provide valuable information for selecting superior feed efficiency meat-type chickens.

In conclusion, three SNPs and an ACA haplotype of the *Pit-1* gene were significantly associated with growth and feed efficiency traits in chickens and might be used as potential molecular genetic markers in yellow meat-type chicken breeding programs. Further researches are needed to properly examine the associations of polymorphisms of *Pit-1* with growth and feed efficiency traits in large populations of distinct chicken strains. In addition, the functional mechanisms of identified SNPs or haplotypes affecting growth and feed efficiency traits should be further investigated.

## Figures and Tables

**Table 1:** Ingredient compositions and analyzed nutrient levels of the experimental diet

Item	Content (%)
Ingredients	
Corn	64.65
Soybean meal	26.84
Soybean oil	4.35
Limestone	1.30
Calcium hydrogen phosphate	1.38
NaCl	0.30
Choline chloride	0.18
Premix1)	1.0
Total	100.0
Nutrient levels	
Metabolizable energy (Kcal/kg)	2,837
Crude protein	20.36
Crude fiber	3.15
Total phosphorus	0.63
Available phosphorus	0.49
Methionine	0.39
Lysine	0.64

1) Premix provided the following per kilogram of diet: vitamin A, 12,000 IU; Vitamin D_3_, 3,000 IU; Vitamin E, 25 IU; Vitamin K_3_ 3.0 mg; Vitamin B_1_, 2.5 mg; Vitamin B_2_, 5.0 mg; Vitamin B_6_ 1.50 mg Vitamin B_12_, 0.020 mg; nicotinic acid 0.20 mg; folic acid 0.75 mg; pyridoxine 4.0 mg; NaHCO_3_ 1,000 mg; Fe, 80 mg; Zn, 60 mg; Cu, 8 mg; Mn, 50 mg; I, 0.40 mg; Se, 0.10 mg.

**Table 2:** Primer sequences used in genotyping for the *Pit-1* gene

SNP	Primer	Sequence (5′ to 3′)
rs13687126	Forward	ACGTTGGATGTTGAATGTGAGAGGAGAAGG
	Reverse	ACGTTGGATGTCCTCTGTCAATGCCATCTC
	Extension	AGCCCCACAATGACA
rs13687128	Forward	ACGTTGGATGCTCAAGATTAAGCCCCTCAG
	Reverse	ACGTTGGATGGGCACTTTGGAGAACAAAGC
	Extension	AACGCCTTCTTCTCAGGAAAT
rs13905624	Forward	ACGTTGGATGTTTATACGTAGGCACAACAG
	Reverse	ACGTTGGATGTAAAGTGCCCTCTTGTGTCC
	Extension	GCTTAAGCCAGCTTTGG
rs13905622	Forward	ACGTTGGATGCAGCTGCTGAGGAAATCAAG
	Reverse	ACGTTGGATGTAGCCTCAGTGTAATGAGGG
	Extension	TGTAATGAGGGTAGATATAAAAG
rs13905628	Forward	ACGTTGGATGGTCTGAGAGCTACAATCGTG
	Reverse	ACGTTGGATGGAGGGGAATGGTTTTTGTGG
	Extension	GTTGTGGTTTTGTTTTGTTTTGTT

*Pit-1*, pituitary specific transcription factor-1; SNP, single nucleotide polymorphism.

**Table 3:** Genotype and allele frequencies of five SNPs in *Pit-1* gene and Hardy-Weinberg equilibrium test

SNP	Location	Sample	Genotypic frequency			Allelic frequency		p-value for HWE
	
			Major1)	Heterozygous	Minor	Major	Minor	
rs13687126	promoter	710	0.33	0.44	0.23	0.55	0.45	0.1129
rs13687128	exon	704	0.28	0.49	0.23	0.52	0.48	0.4845
rs13905622	exon	695	0.40	0.43	0.17	0.62	0.38	0.2144
rs13905624	exon	696	0.50	0.41	0.09	0.70	0.30	0.9004
rs13905628	exon	690	0.28	0.45	0.27	0.51	0.49	0.1603

SNP, single nucleotide polymorphism; *Pit-1*, pituitary specific transcription factor-1; HWE, Hardy-Weinberg Equilibrium test.

1) Three genotypes of the *Pit-1* gene: Major, heterozygous, and minor.

**Table 4:** Association analysis of *Pit-1* polymorphisms and haplotype with growth and feed efficiency traits in chickens

Growth and feed efficiency traits	p-value for significant test					

	rs13687126	rs13687128	rs13905624	rs13905622	rs13905628	ACA haplotype
BW49	0.4305	0.3423	0.3044	0.1206	0.9029	0.3145
BW70	0.1103	0.0156*	0.8343	0.0178*	0.2344	0.0135*
BWG	0.0275*	0.0450*	0.8675	0.2984	0.2757	0.1288
FI	0.0401*	0.1745	0.5388	0.1458	0.1090	0.0545
FCR	0.1232	0.0253*	0.0688	0.0223*	0.1756	0.0367*

*Pit-1*, pituitary specific transcription factor-1; BW49, body weight at 49 days of age; BW70, body weight at 70 days of age; BWG, body weight gain from 49 to 70 days of age; FI, feed intake from 49 to 70 days of age; FCR, feed conversion ratio from 49 to 70 days of age.

* p<0.05,

** p<0.01.

**Table 5:** Haplotypes based on SNPs of rs13687126, rs13687128, and rs13905622 in the *Pit-1* gene

No.	Haplotype	Observations	Frequency (%)
H1	ACA	703	48.35
H2	ACT	126	8.08
H3	ATA	89	6.01
H4	ATT	147	9.98
H5	GCA	45	3.01
H6	GCT	65	4.41
H7	GTA	276	19.02

*Pit-1*, pituitary specific transcription factor-1.

**Table 6:** Least squares means±SE for growth and feed efficiency traits among genotypes of three SNPs in the *Pit-1* gene1)

SNP	Genotype	Growth and feed efficiency traits				

		BW49 (g)	BW70 (g)	BWG (g)	FI (g)	FCR
rs13687126	AA(232)	1,109.70±2.28	1,715.46±2.37	606.76±1.89^ab^	1,776.83±3.86^a^	2.93±0.01
	AG(313)	1,113.37±5.78	1,714.90±3.02	601.53±2.82^a^	1,766.18±4.81^a^	2.93±0.02
	GG(165)	1,111.55±2.84	1,724.30±2.37	613.74±1.90^b^	1,784.15±3.78^b^	2.91±0.09
rs13687128	CC(195)	1,104.62±3.03	1,705.62±3.12^a^	601.00±2.50^a^	1,774.30±5.09	2.95±0.02^a^
	CT(343)	1,103.56±4.51	1,703.87±4.08^a^	600.08±2.85^a^	1,768.18±6.38	2.94±0.01^a^
	TT(166)	1,108.54±3.40	1,720.50±3.84^b^	612.95±4.58^b^	1,778.09±5.14	2.90±0.02^b^
rs13905622	AA(280)	1,115.65 ±4.35	1,721.92±4.84^a^	606.27±3.28	1,767.07±4.85	2.91±0.01^a^
	AT(298)	1,109.22±3.29	1,709.53±4.56^b^	600.31±3.24	1,764.87±3.66	2.94±0.03^b^
	TT(117)	1,106.56±5.25	1,708.90±3.47^b^	602.34±4.48	1,770.21±4.91	2.94±0.02^b^

SE, standard error; SNPs, single nucleotide polymorphisms; *Pit-1*, pituitary specific transcription factor-1; BW49, body weight at 49 days of age; BW70, body weight at 70 days of age; BWG, body weight gain from 49 to 70 days of age; FI, feed intake from 49 to 70 days of age; FCR, feed conversion ratio from 49 to 70 days of age; SEM, standard error of the mean.

1) Data are summarized as means±SEM.

a,b Among genotypes within each SNP for each trait, means without a common superscript differ significantly (p<0.05).

## References

[1] Patience JF, Rossoni-Serao MC, Gutierrez NA (2015). A review of feed efficiency in swine: biology and application. J Anim Sci Biotechnol.

[2] de Verdal H, Narcy A, Bastianelli D (2011). Improving the efficiency of feed utilization in poultry by selection. 2. Genetic parameters of excretion traits and correlations with anatomy of the gastro-intestinal tract and digestive efficiency. BMC Genet.

[3] Steinfelder HJ, Hauser P, Nakayama Y (1991). Thyrotropin-releasing hormone regulation of human TSHB expression: role of a pituitary-specific transcription factor (Pit-1/GHF-1) and potential interaction with a thyroid hormone-inhibitory element. Proc Natl Acad Sci USA.

[4] Li S, Crenshaw EB, Rawson EJ (1990). Dwarf locus mutants lacking three pituitary cell types result from mutations in the POU-domain gene *pit-1*. Nature.

[5] Lee EJ, Russell T, Hurley L, Jameson JL (2005). Pituitary transcription factor-1 induces transient differentiation of adult hepatic stem cells into prolactin-producing cells i*n vivo*. Mol Endocrinol.

[6] Taha D, Mullis PE, Ibanez L, de Zegher F (2005). Absent or delayed adrenarche in Pit-1/POU1F1 deficiency. Horm Res.

[7] Sobrier ML, Tsai YC, Perez C (2016). Functional characterization of a human POU1F1 mutation associated with isolated growth hormone deficiency: a novel etiology for IGHD. Hum Mol Genet.

[8] Hoseinzadeh ZE, Mohammadabadi MR, Esmailizadeh AK, Khezri A (2015). Association of PIT1 gene and milk protein percentage in Holstein cattle. J Livest Sci Tech.

[9] Piórkowska K, Ropka-Molik K, Oczkowicz M, Różycki M, Żukowski K (2013). Association study of *PIT1* and *GHRH* SNPs with economically important traits in pigs of three breeds reared in Poland. Anim Sci Pap Rep.

[10] Tang LG, Dongying Yang DY, Ouyang WQ (2012). Association of polymorphisms in the *Pit-1* intron 5 with body measurements in Chinese Cattle. Afr J Biotechnol.

[11] McElroy JP, Kim JJ, Harry DE (2006). Identification of trait loci affecting white meat percentage and other growth and carcass traits in commercial broiler chickens. Poult Sci.

[12] Nie Q, Fang M, Xie L (2008). The PIT1 gene polymorphisms were associated with chicken growth traits. BMC Genet.

[13] Xu HY, Wang Y, Liu YP, Wang JW, Zhu Q (2012). Polymorphisms and expression of the chicken *POU1F1* gene associated with carcass traits. Mol Biol Rep.

[14] Jin S, Chen S, Li H (2013). Polymorphisms in the transforming growth factor beta3 gene and their associations with feed efficiency in chickens. Poult Sci.

[15] Barrett JC, Fry B, Maller J, Daly MJ (2005). Haploview: analysis and visualization of LD and haplotype maps. Bioinformatics.

[16] Ardlie KG, Kruglyak L, Seielstad M (2002). Patterns of linkage disequilibrium in the human genome. Nat Rev Genet.

[17] Bona G, Paracchini R, Giordano M, Momigliano-Richiardi P (2004). Genetic defects in GH synthesis and secretion. Eur J Endocrinol.

[18] Cohen LE, Wondisford FE, Radovick S (1996). Role of Pit-1 in the gene expression of growth hormone, prolactin, and thyrotropin. Endocrinol Metab Clin North Am.

[19] Sewalem A, Morrice DM, Law A (2002). Mapping of quantitative trait loci for body weight at three, six, and nine weeks of age in a broiler layer cross. Poult Sci.

[20] Manjula P, Choi N, Seo D, Lee JH (2018). POU class 1 homeobox 1 gene polymorphisms associated with growth traits in Korean native chicken. Asian-Australas J Anim Sci.

[21] Selvaggi M, Dario C, Normannno G, Dambrosio A, Dario M (2011). Analysis of two pit-1 gene polymorphisms and relationships with growth performance traits in Podolica young bulls. Livest Sci.

[22] Bhattacharya TK, Chatterjee RN, Priyanka M (2012). Polymorphisms of Pit-1 gene and its association with growth traits in chicken. Poult Sci.

[23] Song C, Gao B, Teng Y (2005). MspI polymorphisms in the 3rd intron of the swine POU1F1 gene and their associations with growth performance. J Appl Genet.

[24] Sadeghi M, Jalil-Sarghale A, Moradi-Shahrbabak M (2014). Associations of *POU1F1* gene polymorphisms and protein structure changes with growth traits and blood metabolites in two Iranian sheep breeds. J Genet.

