# Supplementary material for: Association of polymorphisms in Pit-1 gene with growth and feed efficiency in meat-type chickens
Source: Asian-Australas J Anim Sci. 2018 Jul 26;31(11):1685–90. doi: 10.5713/ajas.18.0173 (PMC6212749; doi:10.5713/ajas.18.0173)
Supplement: Supplementary file 1 [file ajas-31-11-1685-supplementary.pdf]

Supplementary Table S1. Descriptive statistics of feed efficiency and relevant traits in meat-type chickens

Growth and feed efficiency traits[1]	N202		N301	
	Mean	SEM[2]	Mean	SEM
BW49(g)	1243.67	4.82	976.31	2.78
BW70(g)	2042.98	5.41	1381.88	3.52
BWG(g)	799.32	3.33	405.57	2.24
FI(g)	2420.46	8.77	1127.11	4.80
FCR	3.03	0.01	2.80	0.01

[1] BW49 = body weight at 49 days of age, BW70 = body weight at 70 days of age, BWG = body weight gain from 49 to 70 days of age, FI = feed intake from 49 to 70 days of age, FCR = feed conversion ratio from 49 to 70 days of age

[2]SE: standard error of the mean

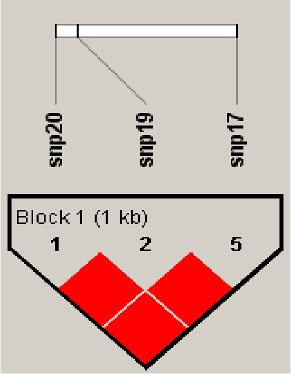

Supplementary Figure S1. The linkage analysis of three SNPs in the *Pit-1* gene. SNP17, SNP19, SNP20 refers to rs13687126, rs13687128, and rs13905622 of the *Pit-1* gene, respectively. The color of block means the LD status of SNPs; red color reveals high linkages among three SNPs.
